# Coordinately express hemicellulolytic enzymes in *Kluyveromycesmarxianus* to improve the saccharification and ethanol production from corncobs

**DOI:** 10.1186/s13068-021-02070-1

**Published:** 2021-11-22

**Authors:** Qing Lan, Yitong Duan, Pingping Wu, Xueyin Li, Yao Yu, Bo Shi, Jungang Zhou, Hong Lu

**Affiliations:** 1 State Key Laboratory of Genetic Engineering, School of Life Sciences, Fudan University, 2005 Songhu Road, Shanghai, 200438 People’s Republic of China; 2 Shanghai Engineering Research Center of Industrial Microorganisms, Fudan University, 2005 Songhu Road, Shanghai, 200438 People’s Republic of China; 3 Shanghai Collaborative Innovation Center for Biomanufacturing (SCICB), East China University of Science and Technology, 130 Meilong Road, Shanghai, 200237 People’s Republic of China; 4 Key Laboratory for Feed Biotechnology of the Ministry of Agriculture, Feed Research Institute, Chinese Academy of Agricultural Sciences, 12 Zhongguancun South Street, Beijing, 100081 People’s Republic of China

**Keywords:** *Kluyveromycesmarxianus*, Hemicellulases, Ribosomes skipping, Enzymatic hydrolysis, Ethanol

## Abstract

**Background:**

Hemicellulose acts as one factor contributing to the recalcitrance of lignocellulose that prevents cellulases to degrade the cellulose efficiently even in low quantities. Supplement of hemicellulases can enhance the performance of commercial cellulases in the enzymatic hydrolyses of lignocellulose. *Kluyveromyce marxianus* is an attractive yeast for cellulosic ethanol fermentation, as well as a promising host for heterologous protein production, since it has remarkable thermotolerance, high growth rate, and broad substrate spectrum etc. In this study, we attempted to coordinately express multiple hemicellulases in *K.* *marxianus* through a 2A-mediated ribosome skipping to self-cleave polyproteins, and investigated their capabilities for saccharification and ethanol production from corncobs.

**Results:**

Two polycistronic genes *IMPX* and *IMPαX* were constructed to test the self-cleavage of P2A sequence from the Foot-and-Mouth Disease virus (FMDV) in *K.* *marxianus*. The *IMPX* gene consisted of a β-mannanase gene *M330* (without the stop codon), a P2A sequence and a β-xylanase gene *Xyn-CDBFV* in turn. In the *IMPαX* gene, there was an additional α-factor signal sequence in frame with the N-terminus of *Xyn-CDBFV.* The extracellular β-mannanase activities of the IMPX and IMPαX strains were 21.34 and 15.50 U/mL, respectively, but the extracellular β-xylanase activity of IMPαX strain was much higher than that of the IMPX strain, which was 136.17 and 42.07 U/mL, respectively. Subsequently, two recombinant strains, the IXPαR and IMPαXPαR, were constructed to coordinately and secretorily express two xylantic enzymes, Xyn-CDBFV and β-D-xylosidase RuXyn1, or three hemicellulolytic enzymes including M330, Xyn-CDBFV and RuXyn1. In fed-batch fermentation, extracellular activities of β-xylanase and β-xylosidase in the IXPαR strain were 1664.2 and 0.90 U/mL. Similarly, the IMPαXPαR strain secreted the three enzymes, β-mannanase, β-xylanase, and β-xylosidase, with the activities of 159.8, 2210.5, and 1.25 U/mL, respectively. Hemicellulolases of both strains enhanced the yields of glucose and xylose from diluted acid pretreated (DAP) corncobs when acted synergistically with commercial cellulases. In hybrid saccharification and fermentation (HSF) of DAP corncobs, hemicellulases of the IMPαXPαR strain increased the ethanol yield by 8.7% at 144 h compared with the control. However, both ethanol and xylose yields were increased by 12.7 and 18.2%, respectively, at 120 h in HSF of aqueous ammonia pretreated (AAP) corncobs with this strain. Our results indicated that coordinate expression of hemicellulolytic enzymes in *K. marxianus* promoted the saccharification and ethanol production from corncobs.

**Conclusions:**

The FMDV P2A sequence showed high efficiency in self-cleavage of polyproteins in *K*. *marxianus* and could be used for secretory expression of multiple enzymes in the presence of their signal sequences. The IMPαXPαR strain coexpressed three hemicellulolytic enzymes improved the saccharification and ethanol production from corncobs, and could be used as a promising strain for ethanol production from lignocelluloses.

## Background

Lignocellulose is the most abundant renewable resource on earth, which is recalcitrance and compact biomass that composes of directly interlinked cellulose, hemicelluloses and lignin. Utilization of lignocellulosic biomass is a feasible solution to avoid excessive reliance on fossil fuels, and alleviates global warming and environmental pollution events [1]. Unlike first-generation biofuels used edible feedstocks, cellulosic ethanol is the second-generation biofuel manufactured from non-edible carbohydrates of plant cell walls [2]. This progress is expected to avert the competition for food and energy demand concurrent with the growth of the world population that has exerted great stress on current agriculture, and provided more environmental benefits as it was carbon–neutral avoiding an increase of greenhouse gases in the atmosphere [3, 4]. Generally, bioethanol is used alone, or mixed in varying amounts with gasoline, and is a more uniform and cleaner source of fuel than the other two biofuels, biodiesel and biogas, that are samely made from living matter. However, both biodiesel and biogas suffer uncertain combustion standardizations because their cetane numbers or gelatinization temperatures highly depend on the source of lipid or the methane content that varies with the substrate composition and digestion method [5]. Bioethanol production is basically composed of four phases that include pretreatment, hydrolysis, fermentation and dehydration. Hydrolysis (saccharification) of pretreated lignocelluloses is a critical prerequisite for ethanolic fermentation by microbes, but usually, it can be concurrently integrated with fermentation, known as simultaneous saccharification and fermentation (SSF), when using enzymes for the hydrolysis [6].

To depolymerize lignocelluloses into fermentable sugars, at least three types of cellulases, such as β-1,4-endoglucanase, exocellobiohydrolase, and β-1,4-glucosidase, and a remarkable diversity of hemicellulases including β-1,4-xylanase, β-1,4-xylosidase, β-1,4-mannanase, α-arabinosidases, esterases, etc., are required to act synergistically. But the less catalytic efficiency and high cost of enzymes made the cellulose hydrolysis become the major bottleneck for bringing down the production cost of biofuel from lignocelluloses [7, 8]. A consolidated bioprocessing (CBP) strategy that integrated enzyme production, saccharification, and fermentation in one step is well accepted as an attractive approach to reduce the cost of biofuel production [9]. Despite the conventional yeasts and bacteria for separated hydrolysis and fermentation (SHF) and SSF processes are well established, the use of ideal CBP is still on the way. Recently, a hybrid saccharification and fermentation (HSF), also called hybrid hydrolysis and fermentation (HHF), is set out by hydrolyzing pretreated lignocelluloses with cellulases before a CBP or SSF process [10, 11].

CBP microbes for cellulosic ethanol were genetically modified from either natural cellulolytic bacteria (*Cellulolytic thermophiles*, *Thermoanaerobacterium saccharolyticum*, *Caldicellulosiruptor bescii*, etc.) and filamentous fungi (*Trichoderma reesei*, *Aspergillus niger,* *Fusarium oxysporum*, and *Penicillium oxalicum* etc.), or ethanologenic microorganisms including *Saccharomyces cerevisiae*, *Kluyveromyces* *marxianus,* *Zygosaccharomyces bailii* and *Zymomonas mobilis,* that have combined the cellulase production, enzymatic hydrolysis, and microbial fermentation into a single operation [12]. But the low ethanol tolerance is an actual inferiority for cellulolytic microbes, since distillation of ethanol is an energy-intensive process and it consumes more of heat to separate ethanol from a lower concentration fermentation [12, 13]. Co-fermentation of pentoses, xylose and arabinose, is a reasonable way to raise the bioethanol concentration from lignocellulosic biomass and reduce the cost of cellulosic ethanol at the same time [14]. As the most utilized yeast for ethanol fermentation, *S. cerevisiae* is unable to assimilate xylose and other C5 sugars, which impedes the efficient ethanol conversion from lignocellulose even it has high ethanol productivity and tolerance [15]. *K. marxianus* is regarded as another attractive yeast for ethanolic fermentation due to its abilities of fastest growth, remarkable thermotolerance, and broad substrate spectrum including glucose, mannose, galactose, lactose, cellobiose, the pentose sugars xylose and arabinose that are virtually presented in all enzymatic hydrolysates of pretreated lignocelluloses [16–18]. Factually, either in SSF or HSF, high-temperature fermentation can significantly elevate the efficiency of lignocellulose hydrolysis, decrease the risk of contamination, and curtail the ethanol production phase [6].

To be ethanologenic CBP strains, enzymes responsible for cellulose hydrolysis are required to simultaneously express in one host, while the genetic basis of *K. marxianus* is less well understood [19]. A synthetic biology technique termed “Promoter-based Gene Assembly and Simultaneous Overexpression (PGASO)” was developed to integrate gene cassettes into the *K. marxianus* KY3 genome in a single step, with each gene expression regulated by an individual promoter along with a terminator [20]. In *K. marxianus*, however, the frequency of double homologous recombination is very low, even flanked with long homologous fragments [21]. On the contrary, it has a high activity of non-homologous end-joining (NHEJ) that can efficiently integrate non-homologous DNA fragments into chromosomes via fusing two DNA strands together in the absence of specific sequences [22, 23]. This feature is disadvantageous to integrate expression cassettes into the specific target loci. Herein, we incorporated a different way for co-expression of multiple hemicellulases in one replicative plasmid using a P2A self-processing peptide from foot-and-mouth disease virus (FMDV) in *K. marxianus*. P2A sequences are relatively short oligopeptides located between the P1 and P2 proteins in some *picornavirus* viruses. It can undergo an enzyme-independent self-cleavage at its own C-terminus during protein translation, enabling the ribosome skipping to the next codon to continue the translation [24–26]. By assembling a β-mannanase M330 gene and a β-xylanase Xyn-CDBFV gene into a single ORF with the FMDV P2A [27], the efficiency of P2A self-cleavage in secretory expression of multiple enzymes in *K. marxianus* was evaluated. Subsequently, three hemicellulolytic enzymes were secreted coordinately using the FMDV P2A. In HSFs of corncobs, saccharification and ethanol production were improved when using the engineered strain as a fermentation starter. Our findings demonstrate that the 2A-mediated ribosomes skipping is a good tool for secretory co-expression of multiple enzymes in *K*. *marxianus*, which is greatly beneficial to the construction of CBP strains for cellulosic ethanol production.

## Results and discussion

### Self-cleavage of polyprotein with FMDV P2A in *K. marxianus*

**Fig.1:**
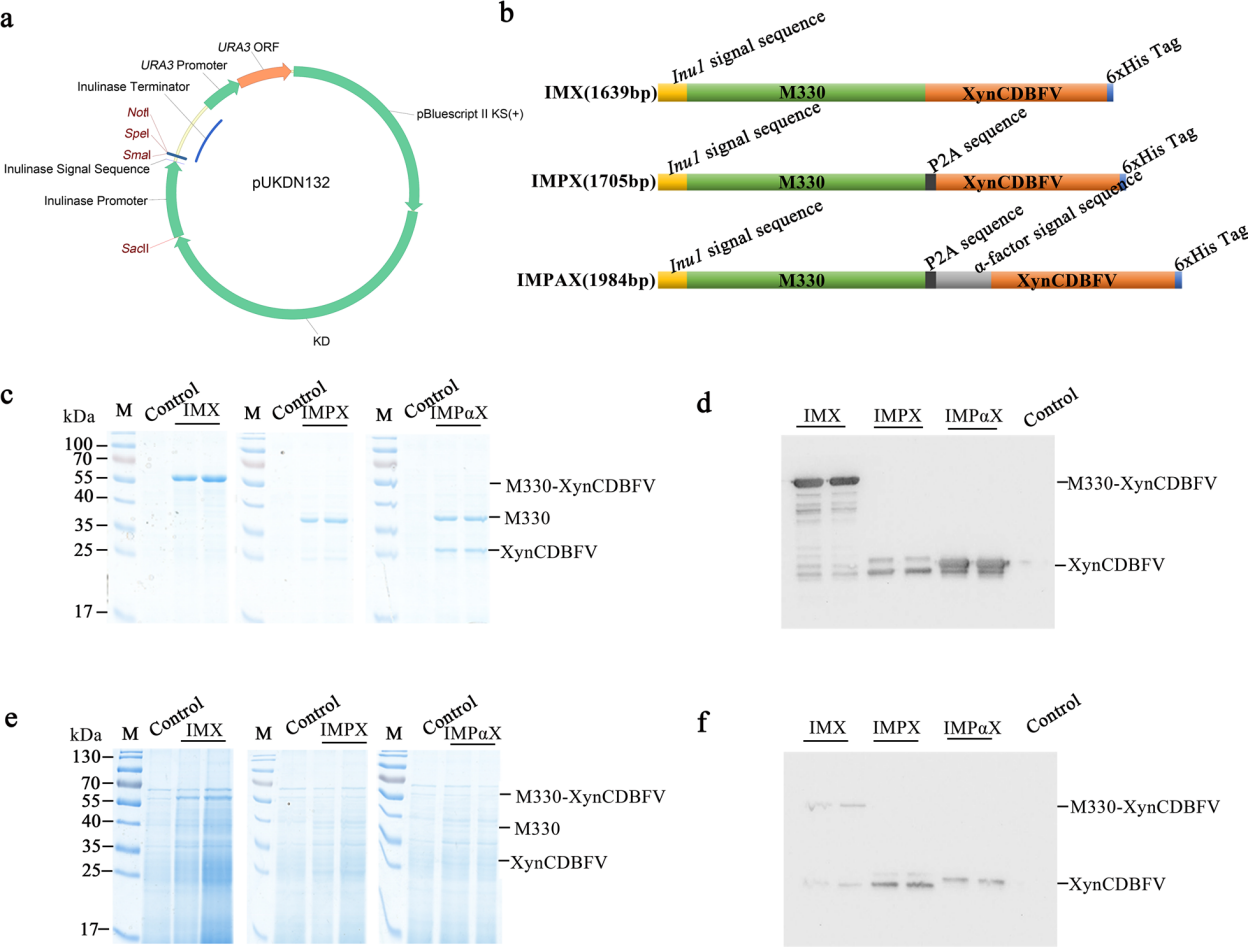
The efficiency of FMDV P2A in self-cleavage of M330 and XynCBDFV. **a** Map of the expression vector pUKDN132; **b** Illustrations of the polycistronic genes *IMX*, *IMPX*, and *IMPαX*; SDS-PAGE and western blots of the supernatants **c**, **d** and cell lysates **e**, **f** of flask cultures

Due to the chemical diversity of hemicellulose structure that heterogeneous polysaccharides with both linear and branched molecules are cross-linked to cellulose microfibrils, complete degradation requires multiple hemicellulases to act synergically [28]. Aiming to facilely express multiple enzymes in ethanologenic *K. marxianus* for the hemicellulose degradation, we resorted to a 2A-mediated ribosome skipping for co-translational cleavage of the polyprotein. In eukaryotic cells, the 2A-mediated cleavage is a common phenomenon that it skips the glycyl–prolyl peptide bond synthesis at the C-terminus of 2A, releases the nascent protein, and resumes the downstream translation [29]. While the 2A self-cleavage efficiency strongly relies on the sequence contexts of upstream and downstream ORFs in the polycistrons [30]. Given that we first tested the efficiency of FMDV P2A self-cleaving in *K. marxianus* by expression of three polycistronic genes *IMX*, *IMPX*, and *IMPαX* (Fig. 1a, b). The *IMX* gene consisted of an M330 coding sequence (*INU1* signal peptide + mature protein coding sequence) and a C-terminal 6xHis-tagged Xyn-CDBFV mature protein coding sequence fused in-frame directly. In the *IMPX* gene, the P2A sequence was incorporated between M330 and Xyn-CDBFV without a stop codon. The *IMPαX* gene had an extra α-factor signal sequence between and Xyn-CDBFV besides the P2A sequence. These three polycistronic genes were cloned into the vector pUKDN132, respectively, in which their expressions were all driven by an *INU1* promoter from *K. marxianus*.

After cultured in flasks, expressions of M330 and Xyn-CDBFV were detected by measuring the activities of β-mannanase and β-xylanase in both supernatants and cell lysates of the IMX, IMPX, and IMPαX strains, obtained by transforming with the plasmids pUKDN132/IMX, pUKDN132/IMPX, and pUKDN132/IMPαX, respectively. Unexpectedly, the IMX strain, as a control, produced high activities of both β-mannanase and β-xylanase in the supernatant, with approximately 24.03 and 155.26 U/mL respectively (Table 1). This result suggested that M330 and Xyn-CDBFV fused directly did not impair their catalytic activities, and its double activities provided a good reference to assessing the effect of P2A on the expression of downstream Xyn-CDBFV. Extracellular β-mannanase activities of the IMPX and IMPαX strains were about 21.34 and 15.50 U/mL, respectively, which were slightly lower than that of the IMX strain. Instead, their intracellular β-mannanase activities were higher than that of the control strain, inferring that fusion of Xyn-CDBFV to the C-terminus of M330 with P2A slightly decreased the secretory expression of M330.

**Table 1:** The β-mannanase and β-xylanase activities of the IMX, IMPX, and IMPαX strains cultured in flasks at 30 °C, 220 rpm for 72 h

Strains	β-Mannanase activities		β-Xylanase activities	
	Extracellular	Intracellular	Extracellular	Intracellular
IMX	24.03 ± 3.74	1.13 ± 0.19	155.26 ± 4.24	44.17 ± 4.24
IMPX	21.34 ± 1.37	4.50 ± 0.75	42.07 ± 4.99	87.59 ± 11.41
IMPαX	15.50 ± 1.91	4.62 ± 0.44	136.17 ± 15.34	39.43 ± 4.11

In our constructs, the efficiency of FMDV P2A self-cleavage was closely associated with the production of Xyn-CDBFV. The IMPαX strains secreted 136.17 U/mL β-xylanase into the supernatants, but retained 39.43 U/mL intracellularly. By contrast, the β-xylanase activity in the supernatant of the IMPX strain was 42.07 U/mL, which was far less than the intracellular activity 87.59 U/mL. To confirm whether the β-xylanase activities of both IMPX and IMPαX strains were the self-cleavaged Xyn-CDBFV by the 2A-mediated ribosomes skipping during translation, these samples were further analyzed by SDS-PAGE and western blot. As shown in Fig. 1c, e, there was a protein band with approximate 57 kDa molecular weight in the supernatants of IMX strain, which was in accordance with the theoretical prediction of the fused IMX protein. In both supernatants of the IMPαX and IMPX strains, M330 and Xyn-CDBFV were secreted alone, but the secretory Xyn-CDBFV of the IMPαX strain was much higher than that of the IMPX strain, suggesting that, in the presence of P2A and α-factor signal sequence, Xyn-CDBFV could be secreted to medium more efficiently. This result was in agreement with the previous literature [31]. Furthermore, western blot assays for the His-tagged Xyn-CDBFV in the above samples were in compliance with the enzymatic assays and SDS-PAGE above (Fig. 1d and f). Nevertheless, to extracellularly express two proteins via FMDV P2A self-cleavage, an extra signal sequence should be included at the N-terminus of the downstream gene. Compared with the internal ribosomal entry site (IRES), which is first identified in *encephalomyocarditis* virus, the 2A-mediated ribosomal 'skipping' is more attractive as it can express multiple cistrons at equimolar levels theoretically [32]. However, it slightly decreased the total level of expressed proteins, especially for the downstream one. This is likely due to the long length of the *IMPαX* gene because gene length is an important regulator for ribosome recruitment and protein synthesis [33]. In *S. cerevisiae*, a ‘long’ gene increased even with a 0.3 kb fragment reduces its transcription clearly [34].

### Coexpression of hemicellulolytic enzymes with FMDV P2A

**Fig.2:**
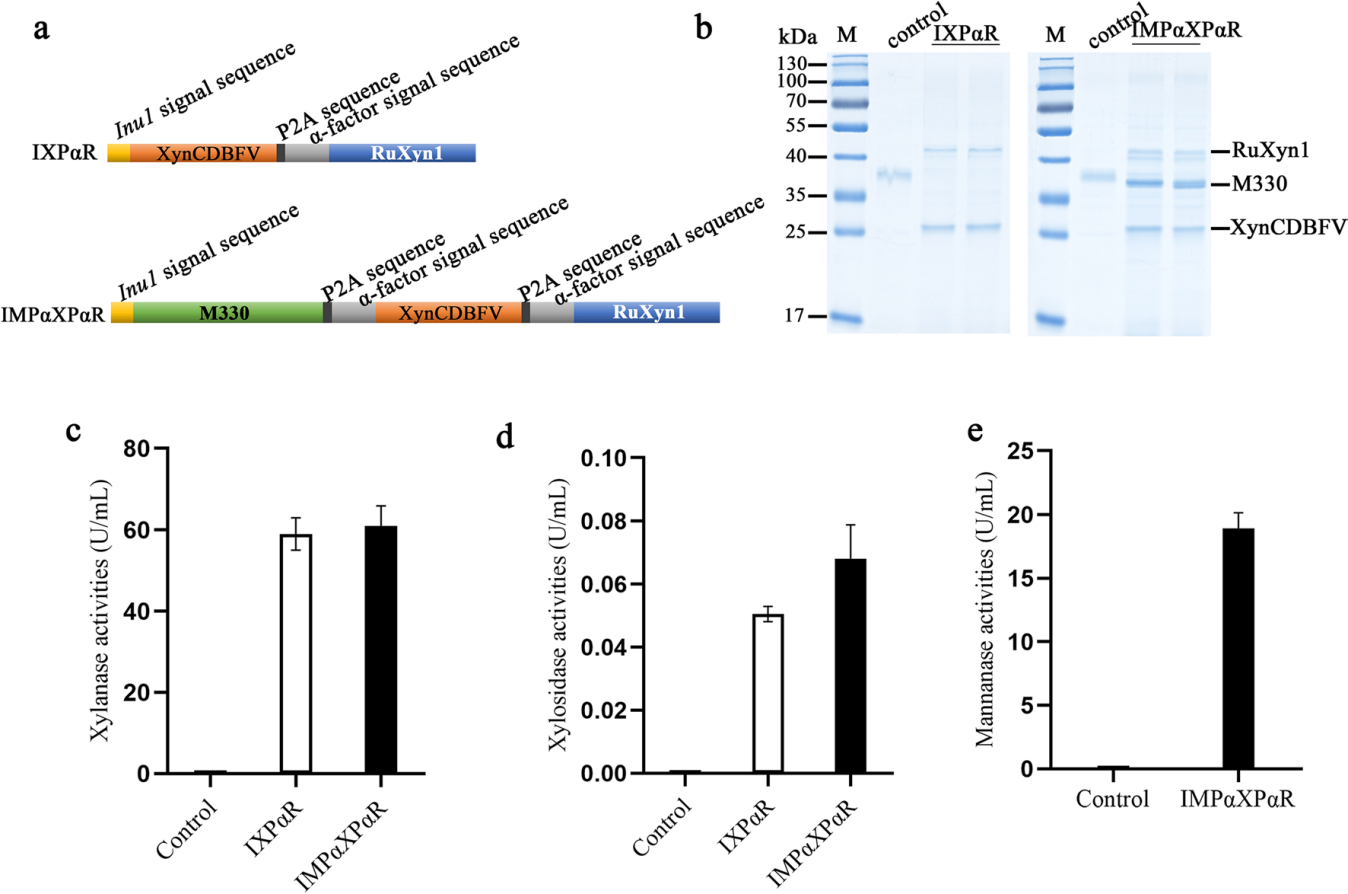
Secretory expression of hemicellulolytic enzymes in *K*. *marxianus*. **a** Constructions of the polycistronic genes *IXPαR* and *IMPαXPαR*; SDS-PAGE **b** and activities of β-xylanase (**c**), β-xylosidase (**d**), and β- mannanase **e** for the supernatants of the IXPαR and IMPαXPαR strains

Hemicellulose acts as one important factor contributing to the recalcitrance of lignocellulose, and they, even in low quantities, can prevent cellulases to degrade cellulose efficiently [35]. Cellulase supplemented with endoxylanase promotes the hydrolysis of steam-exploded feed stocks, releases more glucose, accumulates higher content of xylobiose and xylo-oligosaccharides [36, 37]. Xylose yield, however, was not significantly elevated, which may be due to the insufficient β-xylosidase in most cellulase enzymes produced by filamentous fungi *T. reesei* [38, 39]. Presumably, an ethanologenic strain that co-expresses multiple hemicellulases, especially β-xylanase and β-xylosidase, is able to eliminate the accumulation of xylo-oligosaccharides and produce more fermentable xylose. To test that, a β-xylosidase RuXyn1 with a high capability of xylose conversion from intermediate xylo-oligosaccharides was applied to co-express with β-xylanase in *K. marxianus* [40]. The RuXyn1 coding sequence was fused to Xyn-CDBFV with a P2A and an α-factor signal sequence (Fig. 2a), and then the resulting IXPαR was expressed in *K. marxianus* under the unique *INU1* promoter. The IXPαR strain transformed with the pUKDN132/IXPαR produced 59.01 and 0.05 U/mL of extracellular β-xylanase and β-xylosidase in flask cultures that were grown in YG mediums at 30 °C, 220 rpm for 72 h, respectively (Fig. 2b–d).

Supplements of β-mannanase facilitated the total enzymatic hydrolysis of lignocellulose feedstock and brewery’s by-product, such as beech sawdust, spruce, Douglas fir wood and chips spent grain [41–44]. Given the critical roles of β-mannanase, β-xylanase and β-xylosidase in the hydrolysis of lignocellulose, the feasibility of P2A for coordinately expressing three selected enzymes in one ORF was further tested. A polycistronic gene *IMPαXPαR* compacted *M330*, *Xyn-CDBFV* and *RuXyn1* into one ORF was constructed, each with a signal sequence (Fig. 2a). Consistent with the IMPαX and IXPαR strains, activities for all three enzymes were detectable in the crude supernatant of the IMPαXPαR strain obtained by transformation of the pUKDN132/IMPαXPαR plasmid. Specifically, the activities of β-mannanase, β-xylanase and β-xylosidase were 18.90, 61.00, and 0.07 U/mL, respectively, after culture in YG mediums at 30 °C, 220 rpm for 72 h (Fig. 2c–e). As expected, Fig. 2b showed three protein bands in the culture supernatant of the IMPαXPαR strain corresponding to the predicted molecular weights of M330, Xyn-CDBFV and RuXyn1, which confirmed that FMDV P2A was applicable for secretory co-expression of multiple enzymes in *K*. *marxianus*.

### Preparation of hemicellulolase mixtures by recombinant *K. marxianus* strains

**Fig.3:**
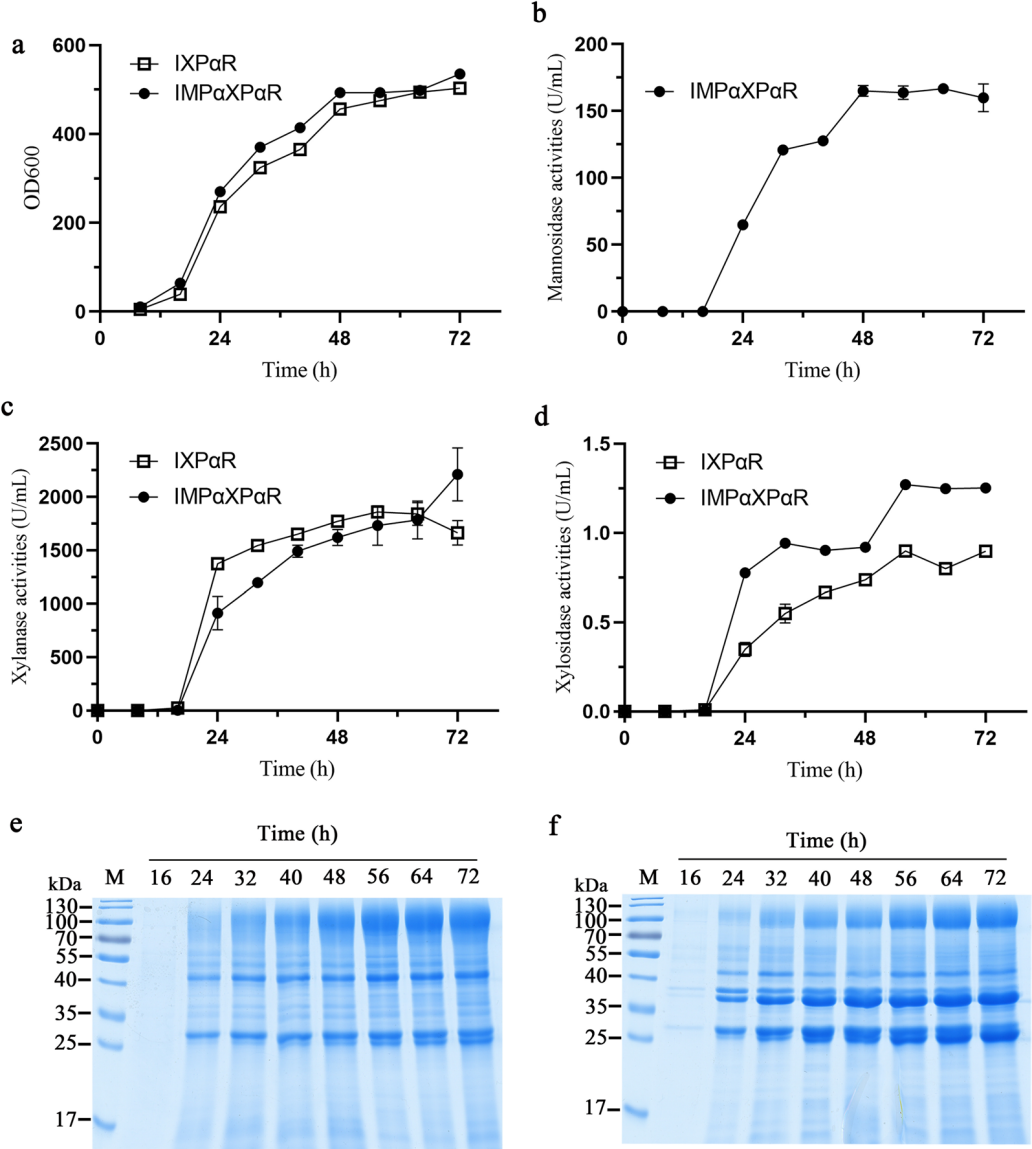
Growth curves **a** and productions of β-mannanase (**b**), β-xylanase (**c**), and β-xylosidase **d** in fed-batch fermentation of the IXPαR and IMPαXPαR strains. Supernatant samples at the indicated times of the IXPαR **e** and IMPαXPαR **f** strains were also analyzed by SDS-PAGE

We have previously developed a high-cell density fed-batch fermentation for single hemicellulolytic enzyme production in *K. marxianus* [18]. In this study, the productions of multiple enzymes in fed-batch fermentation for both the IXPαR and IMPαXPαR strains were also evaluated. *K. marxianus* is a Crabtree-negative yeast that does not perform aerobic alcoholic fermentation, but can respire even in high glucose concentrations [45]. However, high glucose concentration adversely causes respiratory repression and turn to alcoholic fermentation especially in high-cell density, probably due to the insufficient oxygen supply. Similar to *S. cerevisiae*, a Crabtree positive yeast that predominantly produces ethanol in high glucose even in sufficient oxygen levels, it is practicable to guide *K. marxianus* to utilize glucose for respiratory metabolism and convert carbon resources into cell biomass, as glucose can be fed slowly to maintain a concentration below the threshold value in fed-batch fermentation [46, 47]. Additionally, ethanol fermentation could affect the cell growth of *K. marxianus*, thus decreasing the expression of heterologous proteins. To circumvent this, we controlled the dissolved O_2_ above 10% by limiting the fed rate of glucose during fermentation. The cell densities of both strains reached more than 450 (OD_600nm_) after 48 h (Fig. 3a). It seemed that the production of secretory proteins synchronized with the cell growth, as all enzymes were dramatically accumulated during the stage from 16 to 48 h (Fig. 3b–d). After 72 h, the IXPαR strain secreted 1664.2 U/mL of l β-xylanase and 0.90 U/L β-xylosidase, which were about 28 and 18 folds that of in the flask cultures respectively. SDS-PAGE showed that the IXPαR strain secreted two different protein bands that were the mature forms of Xyn-CDBFV and RuXyn1. The IMPαXPαR strain produced 2210.5 U/mL of β-xylanase and 1.25 U/mL of β-xylosidase, slightly higher than that of the IXPαR strain. As well, this strain also produced 159.8 U/mL of β-mannanase concurrently, and all enzymes were secreted extracellularly as their mature forms (Fig. 3e, f).

### Enzymatic hydrolyses of pretreated corncobs

**Fig.4:**
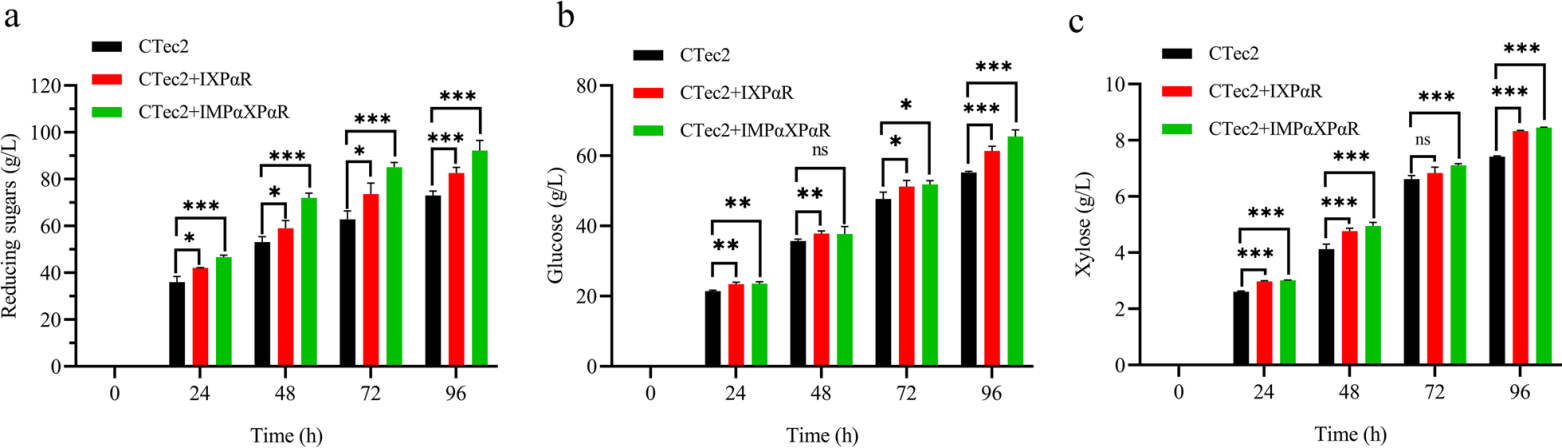
Concentrations of the reducing sugars (**a**), glucose (**b**), and xylose (**c**) over time in the hydrolysis of DAP corncobs. The statistical differences were analyzed using the t-test. *P < 0.05; **P < 0.01; ***P < 0.001

Hemicellulases acted with cellulase significantly enhance the hydrolysis of lignocellulose [48, 49]. The performances of the prepared hemicellulase cocktails on the promotion of lignocellulose hydrolyses were evaluated next using corncob as a feedstock for the enzymatic hydrolyses, because it is one of the most abundant inedible agricultural residues and consists of relatively high content of hemicellulose (∼40%) [50]. Enzymatic hydrolyses were conducted with 10% (w/v) corncobs pretreated by aqueous dilute acid, and 5 FPU of Cellic® CTec2 cellulase per gram solids. After 96 h, about 405.6 mM soluble sugars were released from the pretreated corncobs by Cellic® CTec2 alone. To test the β-xylanase Xyn-CDBFV and β-xylosidase RuXy1 performances on the enzymatic hydrolyses, 300 μl of supernatant collected from the IXPαR strain fed-batch culture at 48 h, equal to 172.7 U β-xylanase and 0.129 U β-xylosidase quantified at 30 ℃, was supplemented to the Cellic® CTec2 cellulase. In accord with previous literatures on pine kraft pulp and softwood [42, 51], supplementations of xylanolytic enzymes to the Cellic® CTec2 cellulase improved the enzymatic hydrolysis of corncobs. At each sampling point, the addition of the enzymes produced by the IXPαR strain generated higher contents of soluble sugars. After hydrolysis for 96 h, soluble sugars increased by 15.7% compared to the Cellic® CTec2 cellulase alone (Fig. 4a). Similarly, the amounts of monomeric glucose and xylose increased to 61.39 and 8.32 g/L, respectively, which were 11.2 and 11.1% higher than that of Cellic® CTec2 cellulase alone (Fig. 4b, c).

The role of β-mannanase M330 for the corncob hydrolysis was also evaluated in combination with β-xylanase and β-xylosidase. The culture supernatant of IMPαXPαR strain containing 8.91 U β-mannanase, 157.85 U β-xylanase, and 0.164 U β-xylosidase quantified at 30 ℃ was supplemented to the Cellic® CTec2 cellulase. As shown in Fig. 4a, the amounts of total soluble sugars were increased over time by the supplementary β-mannanase. At 96 h, about 12.1% more soluble sugars were obtained comparing to that of the IXPαR strain. The glucose and xylose contents were increased to 65.48 and 8.45 g/L (Fig. 4b, c), which were 11.9% and 11.4% higher than that of the xylanolytic enzymes respectively, showing that β-mannanase could facilitate a more extensive break-down of corncobs. This promotion may be ascribed to the deep hydrolysis glucomannan by the synergistic action of β-mannanase with endoglucanase TrCel5A of *T. reesei* presented in Cellic® CTec2, a crude cellulase produced by the ascomycete fungus *T*. *reesei*, since TrCel5A has minor hydrolytic activity towards glucomannans [42].

### HSFs of ethanol from pretreated corncobs

Besides applications in the expression of heterologous proteins, the *K. marxianus* strain used in this study can produce ethanol from multiple substrates, including glucose, xylose, lactose, and inulin, with a maximum ethanol concentration higher than 100 g/L [52, 53]. Enzymatic hydrolyses of the pretreated corncobs indicated that hemicellulolases expressed by the IMPαXPαR strain would be conducive to ethanol production from pretreated lignocellulosic biomass. Subsequently, we evaluated the potential of recombinant IMPαXPαR strain as a fermentation starter to produce ethanol from both the DAP and AAP corncobs. HSFs were performed by pre-hydrolyzing pretreated corncobs with 10 FPU commercial cellulase per gram solids at a solid‐to‐liquid (S/L) ratio of 1:10 (irrespective of the moisture content) for 72 h before being inoculated with the IMPαXPαR or FIM-1 (control) strain. Considering that *K. marxianus* is more strictly Crabtree negative than the model organism species *Kluyveromyces lactis* and other known Crabtree-negative yeasts, it cannot grow under strictly anaerobic conditions and its ethanol fermentation exclusively relies on oxygen limitation [16]. As described previously, dissolved oxygen tension is a key factor for the production of inulinase in *K. marxianus* [54, 55]. In this study, expressions of the hemicellulolytic enzymes in the IMPαXPαR strain were samely driven by the inulinase promoter. To ensure that the hemicellulolytic enzymes were highly expressed and sufficient for HSFs, HSFs starters were prepared by high-cell-density fed-batch culture in 5L fermenters under aeration and agitation. In addition, the effect of the pretreatment mode on the structure and composition of corncobs were also taken into account [56], since it may affect the performance of IMPαXPαR strain in HSFs. Accordingly, corncobs pretreated by the diluted acid and aqueous ammonia, containing 57.4 and 48.9% of glucan, and 8.2 and 32.1% of xylan, respectively, were both used for HSFs.

**Fig.5:**
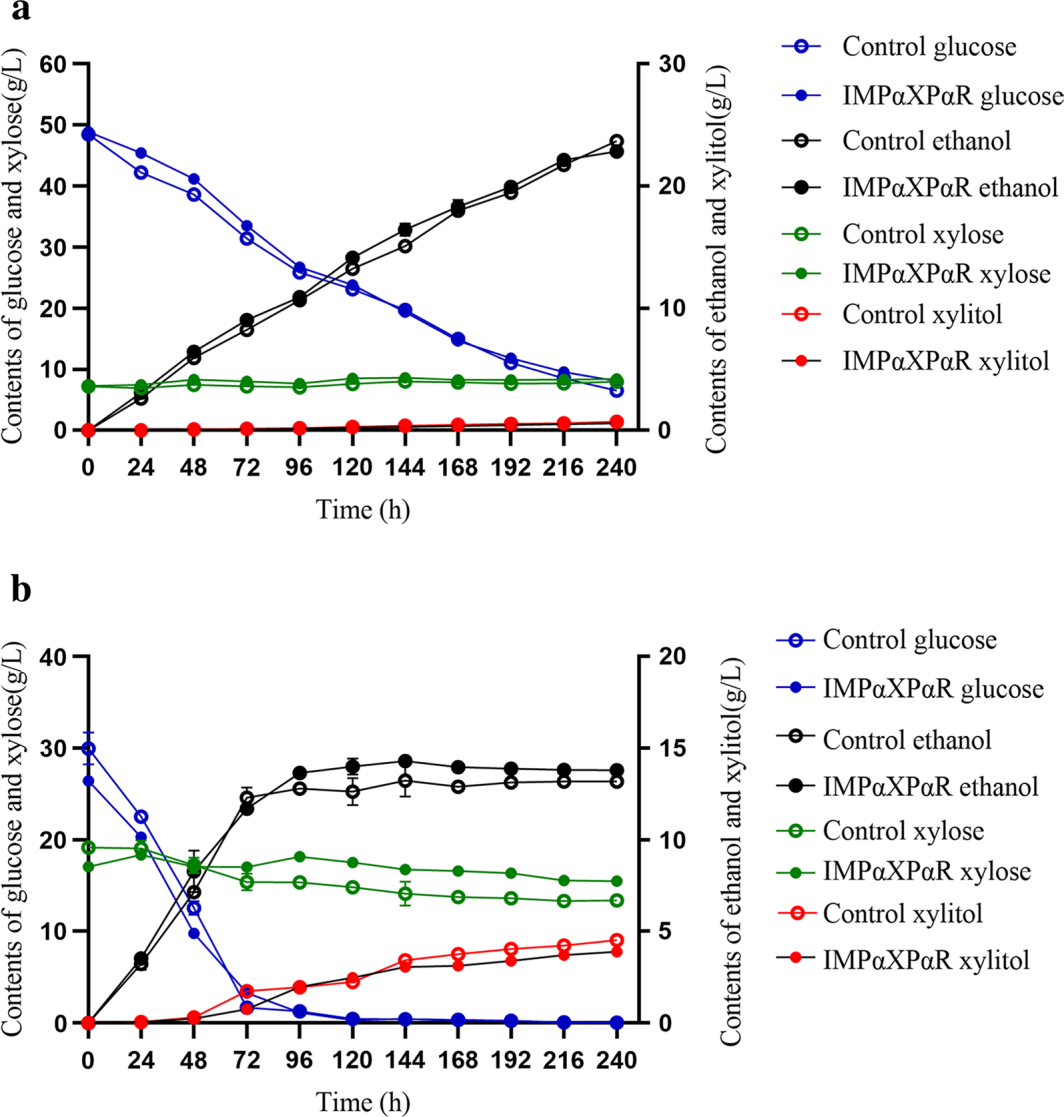
Profiles of glucose, xylose, ethanol, and xylitol in HSFs of the diluted acid **a** and aqueous ammonia **b** pretreated corncobs

After prehydrolysis with Cellic® CTec2 for 72 h, about 80% glucan and 84% xylan of DAP corncobs were degraded into monosaccharides, liberating 48.5 g/L glucose and 7.2 g/L xylose. Ethanolic fermentation was started by inoculating the cell cultures of the IMPαXPαR or control strains prepared by fed-batch fermentation. As shown in Fig. 5a, contents of glucose and xylose in HSFs with IMPαXPαR strain were slightly higher than with FIM-1 strain during the first 72 h when using the DAP corncobs. This was in agreement with the enzymatic saccharification described above. Besides, ethanol yields by the IMPαXPαR strain during the same time were slightly higher than the control as well. After 144 h, the ethanol concentration in HSF with the IMPαXPαR strain was 16.4 g/L, and it was about 8.7% higher than the control 15.1 g/L. In the case of xylitol production, there was no significantly difference between the IMPαXPαR HSFs and the control, since its concentrations were very low in all HSFs, which was below 1 g/L even after 240 h fermentation. Probably, it was due to the strong repression of xylose utilization by glucose in simultaneous fermentation of them with *K*. *marxianus* [57].

But in the case of AAP corncobs, only 60–70% glucan and 62–67% xylan were hydrolyzed by Ctec2 before HSFs. Due to lower saccharification of AAP corncobs by the Cellic® CTec2 cellulase, both glucose and xylose contents in the samples for IMPαXPαR HSFs were lower than those in control, which were 26.4 and 17.9 g/L, and 30.9 and 19.4 g/L, respectively. Inevitably, during the preceding 48 h of HSFs, both glucose and xylose contents in the IMPαXPαR HSFs were lower than in the control (Fig. 5b). However, their contents in the IMPαXPαR HSFs became apparently higher than in the control at 72 h, as there were 3.3 g/L of glucose and 16.9 g/L of xylose in the IMPαXPαR HSFs, and 1.68 g/L of glucose and 15.0 g/L of xylose in the control, respectively. After 120 h, glucose was depleted in all HSFs, but the xylose content in the IMPαXPαR HSFs was still 17.5 g/L, which was 18.2% higher than in the control (*p* < 0.01). The reason for this was that *K. marxianus* cannot assimilate xylose to form ethanol under anaerobic condition [58]. Similarly, ethanol yield in the IMPαXPαR HSFs was 14.2 g/L at this time point, which was 12.7% higher than that of the control (*p* < 0.05). Before 144 h, xylitol yield by the IMPαXPαR strain was not different from the control. These results indicated that the hemicellulolytic enzymes of the IMPαXPαR strain improved the hydrolysis and ethanol production in HSFs of pretreated corncobs, especially with high xylan content of feedstocks.

Comparing with HSFs of DAP corncobs, glucose consumption rate was in apparent higher when using AAP corncobs. Additionally, xylose was consumed to form xylitol by both strains before 48 h in HSFs of AAP corncobs, while its consumption did not occur until 144 h in HSFs of DAP corncobs. A recent study demonstrated that the addition of nitrogen increased the fermentative capacity of *K. marxianus* during ethanol production [59]. Thus, the residual ammonia in AAP corncobs might be the critical foctor that was conducive to the glucose uptake and ethanol production and turned to affect the xylose utilization. As an inexpensive and renewable resource in the world, corncob has been commonly explored as feedstocks for the productions of xylitol, bioethanol, butanol, fatty acids, and other chemicals [50, 60]. However, nitrogen-dependent anaerobic bioethanol fermentation with *K. marxianus* is not fit to the pretreated corncobs with a very high rate of C/N. Fortunately, municipal wastewater containing very low C/N, with 10–100 mg/L of total nitrogen, may offset the nitrogen gap of *K. marxianus* during anaerobic fermentation, which is beneficial for lower biofuel production costs and biological nitrogen removal [61].

## Conclusions

In this study, we used a 2A-mediated ribosome skipping strategy to coordinately express hemicellulolytic enzymes in *K*. *marxianus,* and investigated the performances of the multiple expressed enzymes in saccharification and ethanol production from pretreated corncobs. The FMDV P2A showed high efficiency in secretory co-expression of multiple enzymes in *K. marxianus,* and that three hemicellulolytic enzymes, including a β-mannanase M330, a β-xylanase Xyn-CDBFV, and β-xylosidase RuXyn1, were coordinately secreted in the IMPαXPαR strain. Multiple enzymes of the recombinant *K. marxianus* strains increased both glucose and xylose yields from DAP corncobs when acted with the commercial cellulases, indicating that strengthening of the hemicellulolytic activity could improve the enzymatic saccharification of lignocellulose. Considering the effect of oxygen limitation on the expression of multiple enzymes in *K*. *marxianus* during ethanol fermentation*,* as well as a compromise of ethanol productivity with enzyme productions, HSFs of pretreated corncobs were conducted using fed-batch cultures grown under aeration and agitation. Ethanol yield in HSF of DAP corncobs with the IMPαXPαR strain was about 8.7% higher than the control, while it was 12.7% higher when using AAP corncobs. When using DAP corncobs, there was no significant difference in the productions of xylose along with xylitol between the IMPαXPαR and control strain. By contrast, in HSFs of AAP corncobs containing a higher content of xylan, the xylose yield in the IMPαXPαR HSFs was 18.2% higher than in the control at 120 h, suggesting that promotions on ethanol and xylose yields in HSFs by hemicellulases were closed to the content of hemicellulose in feedstocks. Our findings demonstrate that the 2A-mediated ribosome skipping is a good tool for secretory co-expression of multiple enzymes in *K*. *marxianus*, which is greatly beneficial to the construction of CBP strains for cellulosic ethanol production.

## Methods

### Strains and plasmids

The *K*.* marxianus* Fim-1Δ*URA3* is a uracil auxotrophic strain derived from the FIM-1 strain that has been deposited in China General Microbiological Culture Collection Center (CGMCC No.10621). The expression plasmid pUKDN132 was constructed as described previously [18].

### Expression plasmids constructions and transformations

A polycistronic gene *M330-Xyn-CDBFV* (hereafter termed the IMX gene) that a β-xylanase (EC 3.2.1.8) gene *Xyn-CDBFV* from *Neocallimastix patriciarum* was directly fused to the C-terminus of β-mannanase (EC. 3.2.1.78) gene *M330* from *Bacillus* *sp.* N16-5 was constructed as described below. The *M330* gene was amplified from the pZP41plasmid by the primers MF and IMXR1 (Table 2), and the *Xyn-CDBFV* gene was amplified with IMXF and XR from a pET21a/Xyn-CDBFV [62]. After purification with a SanPrep Column DNA Gel Extraction Kit (B518131, Sangon Biotech, Shanghai, China), the two PCR fragments were ligated together by Gibson assembly [63], and then used as a template to amplify the fused hybrid gene *IMX* with the primers MF and XR. The resulting PCR amplicon was ligated with the *Spe*I and *Not*I linearized pUKDN132 by Gibson assembly, and generated the plasmid pUKDN132/IMX.

**Table2:** Primer sequences used in this work

Primers	Sequences
MF	ATGAAGTTAGCATACTCCCTCTTGC
IMXR1	GAACTACAGAAACTTTGTGTAAATACGGTGGATGGTTTGGAG
IMXF	TCCACCGTATTTACACAAAGTTTCTGTAGTTCAGCTTCTC
XR	CTAGTGATGATGATGATGGTGATCACCAATGTAAACCTTTGCGTATGG
IMPR	AGGACCGGGGTTTTCTTCCACGTCTCCTGCTTGCTTTAACAGAGAGAAGT TCGTGGCTCCGGATCCTGTAAATACGGTGGATGGTTTGGA
IMPXF	TGGAAGAAAACCCCGGTCCTCAAAGTTTCTGTAGTTCAGCTTCTCACT
PαF1	GGAAGAAAACCCCGGTCCTATGAGATTTCCTTCAATTTTTACTGCAG
αXR1	GAGAAGCTGAACTACAGAAACTTTGCCCGGGTACGTAAGCTTCAGCCTCT
αXF1	AGAGGCTGAAGCTTACGTACCCGGGCAAAGTTTCTGTAGTTCAGCTTCTC
XPR	ATCACCAATGTAAACCTTTGCGTATG
XPαF	ACGCAAAGGTTTACATTGGTGATGGATCCGGAGCCACGAACTTCTCTC
αR1	ATAGCGTTTCTTAACTTTATCAGCCCCGGGTACGTAAGCTTCAGCCTCT
αRF	TGATAAAGTTAAGAAACGCTAT
RR	CAAAGCTTGCGGCCTTAAGCGGCCGCTTACTCATCCATGCCTTCGATGGTG
IXF	AGACGGTGACCCCGGGACTAGTATGAGATTTCCTTCAATTTTTACTG

Two polycistronic genes, *IMPX* and *IMPαX,* that contained a P2A sequence between *M330* and *Xyn-CDBFV* alone or with an α-factor signal sequence from *S*. *cerevisiae* were also constructed. The *P2A* sequence was added to the 3’ terminus of *M330* by PCR using the primers MF and IMPR. The *Xyn-CDBFV* sequence was amplified by the primer pair IMPXF/XR, and ligated with the *P2A* fused *M330*. After that, the full-length *IMPX* was amplified by the primers MF and XR, and then inserted into the pUKDN132, obtaining the pUKDN132/IMPX plasmid. When assembling the *IMPαX* gene, the α-factor signal sequence was amplified from the plasmid pPIC9 (Invitrogen, USA) using the primers PαF1 and αXR1, and the *Xyn-CDBFV* sequence was amplified by the primers αXF1 and XR from the pET21a/Xyn-CDBFV. Three fragments including the *P2A* fused *M330*, α-factor signal sequence, and *Xyn-CDBFV* were ligated together to assemble the polycistronic gene *IMPαX* as described above. After cloned into pUKDN132, the resulting plasmid was then termed as pUKDN132/IMPαX.

The polycistronic *IMPαXPαR* gene integrated three genes into a single ORF was constructed by assembling the *IMPαX* gene lack of the stop codon TAG, a *P2A*-linked α-factor signal sequence and a β-xylosidase (EC.3.2.1.37) gene *RuXyn1* from uncultured Yak rumen microorganism*.* The *IMPαX* fragment and the *P2A-*linked α-factor signal sequence were amplified from pUKDN132/IMPαX by the primer pairs, MF/XPR and XPαF/αR1, respectively, while the *RuXyn1* fragment was amplified from a pET21/RuXyn1 vector using the primers αRF and RR [40]. Three fragments were ligated by Gibson assembly to get the full length of *IMPαXPαR*. After PCR amplification with the primers MF and RR, the *IMPαXPαR* was inserted into the *Spe*I/*Not*I site of pUKDN132 to obtain the plasmid pUKDN132/IMPαXPαR. The plasmid pUKDN132/IXPαR was constructed by inserting the XPαR fragment, amplified from pUKDN132/IMPαXPαR by the primers IXF and RR, into the *Spe*I/*Not*I site of pUKDN132.

For plasmid transformations, the *K*.* marxianus* Fim-1Δ*URA3* was inoculated in 5 ml YPD medium (1% Yeast Extract, 2% Peptone, 2% Glucose, pH 6.5), and cultured at 30 °C, 220 rpm for 20 h. Yeast cells were collected by centrifugation, and all plasmid transformations were conducted according to the method by Antunes et al. [64]. Transformants were then selected on synthetic defined (SD) plates (pH 5.5) containing 0.67% yeast nitrogen base without amino acids (YNB), 2% glucose, and 2% agar.

### Enzymatic assays

The activity of β-mannanase was determined with 0.5% locust bean gum (G0753, Sigma-Aldrich, USA) in 50 mM acetate buffer pH 5.5 at 68 °C [65]. Quantitative assays of β-xylanase were performed using 1% wheat arabinoxylan (P-WAXYL, Megazyme, Bray, Ireland) buffered with 50 mM acetate pH 5.5 at 50 °C [62]. β-xylosidase activities were measured using *p*-nitrophenyl-β-D-xylopyranoside as we described previously [40]. One unit (U) of enzyme activity was defined as the amount of enzyme releasing 1 μmol of reducing sugars or *p*-nitrophenol per minute.

### Western blot assays

Transformants were grown in YG mediums (2% yeast extract, 4% glucose, pH 6.0) at 30 °C, 220 rpm for 72 h. One milliliter of cultures was harvested and centrifuged for 10 min at 5000 rpm to detect the secretory or intracellular expression of enzymes by western blot. To prepare lysate samples, cells were suspended in 1 mL lysis buffer (50 mM HEPES pH 7.5, 140 mM NaCl, 1 mM EDTA, 1% Triton X-100, 0.1% Na-deoxycholate), and then disrupted by a bead-beater (FastPrep-24, MP, California, USA) at 6 m/s for 2 min with 400 μL acid-washed glass beads (G8772, Sigma-Aldrich, Missouri, USA). Western blots were carried out using an Anti-His Tag antibody (M30111, Abmart, Shanghai, China) and a horseradish peroxidase-conjugated goat-anti-mouse secondary antibody (074–1806, KPL, USA) as described previously [18].

### Fed-batch fermentation

All fermentations were performed in 5 L bioreactors (BXBIO, Shanghai, China) with an initial working volume of 1.5 L as described previously [18]. Inoculum seeds were precultured in Erlenmeyer flasks containing 150 mL YG medium at 30 °C, 220 rpm for 18 h [18]. After sterilization and cooling, the temperatures of the bioreactors were set to 30 °C. Batch fermentations were started by inoculating with 150 mL inoculum seeds. After glucose was completely depleted, concentrated mediums consisting of 600 g/L glucose, 5 mg/L biotin, 100 mg/L calcium pantothenate, and 100 mg/L niacin were fed into the reactors at rates of 20–35 mL/h depending on the dissolved oxygen (DO), which should be maintained above 10%. The pHs were controlled automatically at 5.5 with ammonium hydroxide. Samplings at given intervals were determined for cell densities (OD_600_ nm) and enzyme activities.

### Pretreatment of the corncobs

Corncobs, purchased from Bei Piao Bang Bang Corncob Development Company (Beijing, China), were ground to a particle size range of 0.25–0.45 mm (40–60 meshes). For dilute acid pretreatment, corncobs were immersed in an aqueous solution of 2% diluted sulfuric acid at a solid‐to‐liquid (S/L) ratio of 1:5. The mixtures were autoclaved at 121 °C for 1 h. After neutralization with 0.1 N NaOH, the pretreated corncobs were separated by filtration under vacuum, washed with deionized water, and dried at 80 ℃. Aqueous ammonia pretreatment was performed by soaking corncobs with 15% ammonia in a screw-capped bottle at a solid–liquid ratio of 1:7 at 60 °C for 24 h. After pretreatment, the pretreated corncobs were diluted with four volumes of deionized water, filtrated under vacuum, washed with deionized water until the pH reached around 7.0, and dried at 80 °C. Compositions of pretreated corncobs were determined according to National Renewable Energy Laboratory (NREL) procedures LAP-002 and − 005 [66, 67].

### Enzymatic saccharification and fermentation

Enzymatic saccharifications were performed in 150 mL Erlenmeyer flasks with 2 g pretreated corncobs in 20 mL of 50 mM sodium citrate buffer pH 5.5. The corncobs slurries were autoclaved at 121 °C for 30 min. After addition of 5 FPU CTec2 per g corncob or coupled with 300 μL supernatant of the fed-batch fermentation cultures, flasks were stirred in an air incubator shaker at 45 °C, 150 rpm. At given intervals, hydrolysates were sampled for analyses of sugar.

HSFs were conducted in 150 mL flasks each containing 10 g of the diluted acid pretreated (DAP) corncobs (with 4.5% moisture content) or aqueous ammonia pretreated (AAP) corncobs (with 18.6% moisture content). The corncobs were immersed in 80 mL of 50 mM sodium citrate buffer pH 5.5 and autoclaved at 121 °C for 20 min. Following sterilization, 10 FPU CTec2 per gram corncob was added and enzymatic saccharifications were performed at 45 ℃, 150 rpm [68]. After 72 h, 10 mL of filter-sterilized media (20 g/L KH_2_PO_4_, 20 g/L (NH_4_)_2_SO_4_, 10 g/L MgSO4·7H_2_O, 5 g/L yeast extract, and 1 g/L MnSO_4_) [6] and 1 mL of fed-batch cultures, collected at 48 h and adjusted to equal cell densities (OD_600nm_, 300) with sterile deionized water, were added to the corncobs slurries. Sterile deionized water was supplemented to make 100 mL of the total liquid volume. The flasks were incubated at 30 °C without stirring. Every 24 h, 200 μL liquid from each sample was taken, centrifuged and the supernatants were analyzed for the concentrations of glucose, xylose, xylitol, and ethanol.

### Analytical methods

Reducing sugars were determined by the DNS method [69]. HPLC analyses for glucose, xylose, xylitol, and ethanol were performed using a MetaCarb 87H column (300 × 7.8 mm) (Agilent, USA) with a refractive index detector at 35 °C. Twenty microliters of each sample were injected and eluted with 0.01 N H_2_SO_4_ in water at a rate of 0.6 mL/min for 30 min.

## Data Availability

All data generated or analyzed during this study are included in this published article.

## Abbreviations

SHF: Separate Hydrolysis and Fermentation
CBP: Consolidated bioprocessing
HSF: Hybrid saccharification and fermentation
SSF: Simultaneous saccharification and fermentation
FMDV: Foot-and-Mouth Disease virus
DO: The dissolved oxygen
FPU: Filter paper unit
ORF: Open read frame
IRES: The internal ribosomal entry site
DAP: Diluted acid pretreated
AAP: Aqueous ammonia pretreated
